# Predictive Modeling of the Hospital Readmission Risk from Patients’ Claims Data Using Machine Learning: A Case Study on COPD

**DOI:** 10.1038/s41598-019-39071-y

**Published:** 2019-02-20

**Authors:** Xu Min, Bin Yu, Fei Wang

**Affiliations:** 1 Department of Healthcare Policy and Research, Weill Cornell Medicine, New York, NY USA; 2 Department of Computer Science and Technology, Institute for Artificial Intelligence, Tsinghua-Fuzhou Institute for Data Technology, and Bioinformatics Division, BNRist, Tsinghua University, Beijing, China; 3 American Air Liquide, Newark, DE USA

## Abstract

Chronic Obstructive Pulmonary Disease (COPD) is a prevalent chronic pulmonary condition that affects hundreds of millions of people all over the world. Many COPD patients got readmitted to hospital within 30 days after discharge due to various reasons. Such readmission can usually be avoided if additional attention is paid to patients with high readmission risk and appropriate actions are taken. This makes early prediction of the hospital readmission risk an important problem. The goal of this paper is to conduct a systematic study on developing different types of machine learning models, including both deep and non-deep ones, for predicting the readmission risk of COPD patients. We evaluate those different approaches on a real world database containing the medical claims of 111,992 patients from the Geisinger Health System from January 2004 to September 2015. The patient features we build the machine learning models upon include both knowledge-driven ones, which are the features extracted according to clinical knowledge potentially related to COPD readmission, and data-driven features, which are extracted from the patient data themselves. Our analysis showed that the prediction performance in terms of Area Under the receiver operating characteristic (ROC) Curve (AUC) can be improved from around 0.60 using knowledge-driven features, to 0.653 by combining both knowledge-driven and data-driven features, based on the one-year claims history before discharge. Moreover, we also demonstrate that the complex deep learning models in this case cannot really improve the prediction performance, with the best AUC around 0.65.

## Introduction

Chronic Obstructive Pulmonary Disease (COPD) is one type of obstructive lung disease makes people difficult to breathe. The Global Burden of Disease Study reports a prevalence of 251 million cases of COPD globally in 2016, and it is estimated that 3.17 million global deaths were caused by the disease in 2015^1^. In US it was reported that 21% of the COPD patients got readmitted 30 days after discharge and the cost for these readmissions is 18% higher than those for initial hospital stays^2^. The Centers for Medicare and Medicaid Services (CMS) has set COPD as one of their important target diseases for designing policies to reduce readmissions because of this high prevalence and cost. According to Purdy *et al*.^3^, COPD is an ambulatory care sensitive condition where hospital admission could be avoided by effective interventions in primary or preventative care. The risk factors for COPD readmission remain largely unknown. Retrospective^4^ and prospective^5^ studies have been conducted to investigate COPD readmissions.

In recent years, because of the rapid development of computer software and hardware technologies and wide adoption of electronic medical data systems, more and more health related data such as Electronic Health Records (EHR) and medical claims are becoming readily available. Many computational models have been developed based on these data for predicting the risk of hospital readmission. The LACE index^6^ uses four variables (L ength of stay (L), A cuity of the admission (A), C omorbidity of the patient (C) and E mergency department use in the duration of 6 months before admission (E)) to predict the risk of death or nonelective 30-day readmission after hospital discharge among both medical and surgical patients. Similarly, the HOSPITAL score^7^ uses 7 clinical predictors (which are available in patient EHRs) to identify patients at high risk of potentially avoidable hospital readmission within 30 days. Researchers have also explored pure data-driven machine learning approaches for this problem. For example, Hosseinzadeh *et al*.^8^ investigated the predictability of hospital readmission using classical machine learning methods (e.g., naïve Bayes and decision trees) using the claims data from the provincial hospital system in Quebec, Canada. Cauruana *et al*.^9^ applied generalized additive model to predict the hospital readmission risk of a general cohort with around 400,000 patients, where each patient is represented as a vector of about 4,000 dimensions. Sushmita *et al*.^10^ studied the prediction of all-cause hospital readmission with machine learning methods (support vector machine, decision trees, random forests and generalized boosting machine) using the admission data of patients provided by a large hospital chain in the Northwestern United States. These studies have demonstrated the better potential of machine learning models for hospital readmission prediction comparing to LACE and HOSPITAL score.

Recently, deep learning^11^, as a specific type of machine learning models, has attracted attentions of researchers in various fields (e.g., computer vision, speech analysis and natural language processing) because of their superior performance. Researchers have also explored the potential of deep learning approaches in hospital readmission prediction. For example, Wang *et al*.^12^ developed a cost-sensitive deep learning approach combining Convolutional Neural Network (CNN)^13^ and Multi-Layer Perceptron (MLP)^14^ for readmission prediction. Xiao *et al*.^15^ adapted the TopicRNN approach^16^, which combines probabilistic topic modeling^17^ and Recurrent Neural Network (RNN)^18^ to better capture long-term dependencies in sequences, to predict the readmission risk of heart failure patients. Rajkomar *et al*.^19^ also developed an approach that ensembles three deep learning models to predict the risk of 30-day unplanned readmission.

Despite the initial success, so far there is no comprehensive and systematic investigation on the potential of machine learning models for hospital readmission risk prediction. The goal of this paper is to conduct such a study on COPD patients using their longitudinal claims records. The output of our model is the probability that each patient will be readmitted within 30 days at the time of discharge. We comprehensively examined the performance of traditional machine learning models including logistic regression and variants, random forest, Support Vector Machine (SVM) and Gradient Boosting Decision Tree, as well as deep learning models including MLP, CNN, RNN and variants, using both knowledge and data driven patient features.

## Methods and Materials

This paper aims at conducting a systematic comparative study on the performance of different machine learning models for predicting the hospital readmission risk of COPD patients. Here we characterize a machine learning model as either traditional (non-deep) or deep. A traditional model is typically composed of two major steps, feature engineering^20^ and model building^21^. Feature engineering extracts “good” features from the data that are effective for the model building step. Different from traditional methods, a deep learning model^11^ enjoys an end-to-end learning mechanism, where the feature engineering part is implicitly integrated into the learning pipeline. In the following we introduce these two types of approaches formally.

### Traditional Methods

As we introduced above, there are two major steps in traditional methods: feature engineering and model building.

#### Feature Engineering

Our goal is to predict the risk of hospital readmission, which is defined as a readmission to hospital within 30 days of a prior hospital discharge. Therefore, the prediction is made on the day of hospital discharge. Patient features can be constructed from the medical history prior to the discharge day. Here we categorize the patient features as either knowledge- or data-driven. More specifically, we investigate the following knowledge - driven features.

- HOSPITAL Score^7^. The original HOSPITAL score is aggregated from 7 features from different subdomains, wherein 4 of them are available in our claims data, including the number of procedures performed during hospital stay (HOS_Proc), the number of hospital admissions during the previous year (HOS_NOAD), the number of hospital stays with >=5 days (HOS_LOS), and the index admission type (HOS_Index). We use them as separate dimensions in the patient representation.
- LACE Index^6^. The LACE index is aggregated from 4 features, i.e. Length of stay (days) (L), Acute (emergent) admission (A), Charlson Comorbidity Index (C) and Number of ED visits within six months (E). We use them as separate dimensions in the patient representation.
- Handcrafted Features. In addition to HOSPITAL score and LACE index, we also picked 12 features that could be important to our task, including age, gender, length of stay (LOS), number of admissions in the previous year (NOA), total length of all stays in the previous year (LOAS), number of all kinds of admissions (NOAA, including outpatient admissions), number of different types of index admissions (Index, Index_trans, Index_final, Readm, Readm_trans, Readm_final).

One limitation of our data is that some important patient features, such as the Global Initiative for Obstructive Lung Disease (GOLD) severity grade^22^, are not available, therefore we cannot use them in the predictive modeling process.

The other feature category is data-driven features, which includes the following four different types.

- Diagnosis. The patient diagnosis in our data is encoded with the International Classification of Diseases (ICD-9) codes. Considering the large number of distinct ICD-9 codes, we further investigated three different grouping strategies: (1) First three digits of ICD-9; (2) Clinical Classifications Software (CCS) codes; (3) Hierarchical Condition Category (HCC) codes.
- Procedures. The patient procedure information is encoded with three different coding sources, i.e., CCS codes, Berenson-Eggers Type of Service (BETOS) codes, and revenue codes.
- Pharmacy. The pharmacy/medication information is encoded with National Drug Code (NDC), which we further mapped to the Generic Therapeutic Class (GTC) codes for the sake of dimensionality reduction.
- Locations. We also consider the location where the medical service is provided.
  For all four types of data-driven features, we construct the following representations through the analogy with natural language processing^23^:
- Bag-of-Words (BoW) representation, which counts the frequency of each feature in the feature construction time period.
- boolean Bag-of-Words (bBoW), which just cares about whether or not a specific feature appears in the feature construction time period.
- Term Frequency-Inverse Document Frequency (TFIDF) normalization of the BoW representation^23^, which suppresses the impact of highly prevalent features (which could be non-informative) by weighting the feature counts by the inverse of its popularity (counts) in all patients’ records.

For both knowledge- and data-driven features, we use either one year or full period before the hospital discharge date as the feature construction period (also called observation window). The only exceptions are HOSPITAL score and LACE (which are defined over one year). Table 1 summarizes the dimensions of all features introduced above. In addition to the investigation of different groups of features respectively in the predictive modeling process, we also combine multiple groups of features for training the models to see how they can boost the performance

**Table 1:** Dimensions of different kinds of features.

Feature x		Dimension (one-year history)	Dimension (full history)
Knowledge-driven	HOS	4	—
	LACE	4	—
	hand	12	12
Data-driven	DX	9743	10306
	DX_3dig	1153	1169
	DX_CCS	285	285
	DX_HCC	197	197
	PROC	11193	12009
	PROC_group	399	402
	PHAR	20289	22964
	PHAR_GTC	42	42
	LC	32	33

#### Model Building

After the patient features are constructed, we will feed them into a machine learning model for readmission risk prediction. The following models are considered in this paper: (1) Logistic regression and its variants (with ${\ell }_{1}$ or ${\ell }_{2}$ norm regularizations); (2) Random F orest; (3) Support Vector Machine (SVM)^24^, where we only consider the linear case; (4) Gradient Boosting Decision Tree (GBDT)^25^; (5) Multi-Layer Perceptron (MLP)^14^. We introduce more details of these models below.

- Logistic Regression (LR). Logistic regression is a popular model in applied health service research. It can be used to explain the relationship between one dependent binary variable and one or more independent variables. Mathematically, we model the probability logit (which is the log-odds) of the probability of an event, as a linear combination of predictive variables, i.e., $logit(p(y=\mathrm{1|}{\bf{x}};{\bf{w}}))={{\bf{w}}}^{T}{\bf{x}}$, where $logit(p)=\,\mathrm{log}(\frac{p}{1-p})$. The regression coefficients **w** are usually estimated through the maximum likelihood estimation (MLE) procedure, which is equivalent to minimize the negative total data log-likelihood as ${\bf{w}}={\rm{\arg }}\,{{\rm{\min }}}_{{\bf{w}}}-{\sum }_{i}^{N}\,\mathrm{log}\,p({y}_{i}|{{\bf{x}}}_{i};{\bf{w}})$.
- Logistic Regression with ${\ell }_{1}$ penalty (LR_l1). Sometimes the number of independent variables is large, in which case not every of them is useful. In order to promote model sparsity and pick out variables that really contribute to the prediction, we can add ${\ell }_{1}$ regularization to the negative total data log-likelihood^26^, that is, ${\bf{w}}={\rm{a}}{\rm{r}}{\rm{g}}\,{{\rm{\min }}}_{{\bf{w}}}-{\sum }_{i}^{N}\,\mathrm{log}\,p({y}_{i}|{{\bf{x}}}_{i};{\bf{w}})+\beta ||{\bf{w}}{||}_{1}$. *β* > 0 is the tradeoff parameter.
- Logistic Regression with ${\ell }_{2}$ penalty (LR_l2). We can also add ${\ell }_{2}$ regularization to the negative total data log-likelihood to improve numerical stability in the parameter estimation process, i.e., ${\bf{w}}={\rm{\arg }}\,{{\rm{\min }}}_{{\bf{w}}}-{\sum }_{i}^{N}\,\mathrm{log}\,p({y}_{i}|{{\bf{x}}}_{i};{\bf{w}})+\beta ||{\bf{w}}{||}_{2}$. *β* > 0 is the tradeoff parameter.
- Random Forest (RF)^27^. Random forest is an ensemble learning method, which constructs multiple decision trees (each on a randomly sampled feature set) at the training stage. Their outputs will be aggregated in the prediction stage (usually through majority voting) as the final result.
- Support Vector Machine (SVM)^24^. SVM is a discriminative classifier which constructs a hyperplane to separate the two classes with the maximum margin. In particular, solves the following optimization problem ${\bf{w}}={\rm{\arg }}\,{{\rm{\min }}}_{{\bf{w}}}\,{\sum }_{1}^{N}\,{\rm{\max }}\,\mathrm{(0,1}-{y}_{i}({{\bf{w}}}^{T}{{\bf{x}}}_{i}-b))+\lambda ||{\bf{w}}{||}_{2}$, where **w** is the separation hyperplane.
- Gradient Boosting Decision Tree (GBDT)^25^. Gradient boosting is an ensemble model comprising of a set of weak learners obtained in a stage-wise fashion through the minimization of some differentiable prediction loss using functional gradient descent. For GDBT those weak learners are set to be decision trees.
- Multi-layer Perceptron (MLP)^28^. Multi-layer perceptron is a class of feed-forward artificial neural network. It consists of multiple hidden layers with nonlinear processing units, and is trained with the back-propagation technique.

### Deep Learning Methods

One limitation of all traditional machine learning models we introduced above is that they need to aggregate patient features in the observation window to form patient vectors. This ignores the temporality in patient records, which is usually important in healthcare settings as it indicates the disease progression process. To explore such temporality, we construct a set of deep learning models, specifically Convolutional Neural Networks (CNN)^13^, Recurrent Neural Networks (RNN)^18^ and their variants (e.g., Long-Short Term Memory (LSTM)^29^ and Gated Recurrent Unit (GRU)^30^). In addition, we further incorporate contextual event embedding, time-sensitive modeling and attention mechanism into the model building process to enhance the model performance. The details are explained as follows.

#### Contextual Event Embedding

If we concatenate the claim records for each patient according to their associated timestamps, we can obtain a medical event sequence for each patient. Contextual embedding^31^ is a class of techniques that learn a vector based representation for each event in the sequence, such that each vector encodes the contextual information around its corresponding event. Word2Vec^32^ is one representative contextual embedding technique that learns an embedded vector for each word in a document corpus (each document can be viewed as a word sequence).

Claims data can be analogous to the text data as they contain sequences of medical events, which play a similar role as words in texts. The difference is that each medical event is associated with a concrete timestamp in claims data, which could be critical. For example, two medical events with one day and one year gap can have completely different meanings in healthcare setting. Therefore we investigated the following variants of contextual embedding techniques.

1. Using a time window instead of a context window to generate event contexts.
2. Weighting the event pairs according to the temporal gap between them. Higher weights will be given to temporally closer event pairs
3. Med2Vec^33^, which is a contextual embedding technique that is able to learn both event -level and visit-level representations for longitudinal patient records, where the temporal gap information is appended as an additional dimension in the event/visit vectors.

More details of these methods are provided in Supplementary Materials. In addition to these methods, we also implemented the one-hot embedding model as the baseline. Specifically, let *V* be the number of unique medical events, then the one-hot representation of an event is a *V*-dimensional binary vector with value 1 on the dimension corresponding to the event and all other entries being 0.

#### Time Fusion in Deep Models

In order to conveniently explore the event temporalities in patient claims, we investigated three types of patient representations.

1. *Sequence Representation*. We represent the records for each patient as two sequences, an event sequence and a timestamp sequence, and then treat the prediction problem as a sequence classification problem. Specifically, let *V* be the number of distinct medical events. For any specific patient, we have the event sequence $\langle {c}_{1},{c}_{2},\cdots ,{c}_{L}\rangle$, and the the corresponding timestamp sequence $\langle {t}_{1},{t}_{2},\cdots ,{t}_{L}\rangle$, where ${c}_{i}\in \mathrm{[1,}\,\mathrm{2,}\cdots ,V]$, and ${t}_{1}\le {t}_{2}\le \cdots \le {t}_{L}$. We can apply the contextual event embedding techniques introduced above to embed each event *c*_*i*_ as a vector **w**_*i*_, then the event sequence becomes the vector sequence ${{\bf{w}}}_{1},{{\bf{w}}}_{2},\cdots ,{{\bf{w}}}_{L}$. Then we can incorporate time information using a time weighting layer. Given the timestamp sequence, we can get a temporal weight ${d}_{i}\propto softmax(\lambda \cdot {\rm{\Delta }}{t}_{i})$, where Δ*t*_*i*_ is the temporal gap between *t*_*i*_ and the hospital discharge date when the prediction is made on, *λ* is a time scaling parameter to be learned in the training phase.
2. *Matrix Representation with Regular Time Intervals (MR-RTI)*. In this case, we represent the claims records of each patient as a longitudinal matrix similar to what Wang *et al*.^34^ did. The columns correspond to different medical events, so there are ***V*** columns in total. The rows correspond to regular time intervals. For example, each row could represent a day, a week or a month, depending on the time resolution. The (*i*, *j*)-th entry of this matrix is 1, if the *j*-th event is observed at the *i*-th timestamp in the patient’s claims, and 0 otherwise.
3. *Matrix Representation with Irregular Time Intervals (MR-ITI)*. The MR-RTI representation could be very sparse – if the patient did not pay visit to the clinic on a specific day then he/she will have an all-zero row in the matrix. The MR-ITI representation deletes these all-zero rows in MR-RTI, which greatly reduced the matrix sparsity. However, because the time intervals are no longer regular, we also need to record the exact timestamp for each row in the matrix. This is similar to the sequence representation.

**Figure 1:**
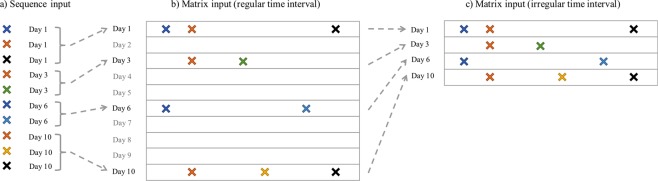
Three types of patient representations for incorporating the temporal information. (**a**) Sequence Representation; (**b**) Matrix Representation with Regular Time Intervals (MR-RTI); (**c**) Matrix Representation with Irregular Time Intervals (MR-ITI).

We summarize the three different patient representations in Fig. 1.

#### Attention Mechanism

In addition to the time weighting layer to incorporate timestamp information, we can also apply attention mechanism on the event embeddings to emphasize more on the important medical events. The attention weight for event *c*_*i*_ is computed using a softmax function ${a}_{i}\propto softmax({\beta }^{T}{{\bf{w}}}_{i})$, where *β* is a reference vector to be learned from the model training process, and **w**_*i*_ is the embedded vector of *c*_*i*_. This attention weight *a*_*i*_ tells us how much attention we should pay on event *c*_*i*_. We can multiply it with the time weight to get a composite weight for each event in the modeling process.

The overall architecture of the deep learning models we investigated is provided in Fig. 3 in the supplemental material.

## Results

The detailed experimental results are presented in this section. First we introduce the process of data preprocessing.

### Data Preprocessing

Our raw data contain 111,992 patients in Geisinger Health System who had at least one COPD related diagnosis (ICD-9 diagnosis codes: 490.**, 491.**, 492.**, 493.2*, 494.**, 496.**) between January 2004 and September 2015. The information contained in patient claims include patient demographics, medication, service location (utilization), diagnosis and procedure. Table 1 in the supplemental material summarizes the details of each type of information.

We built a three-step pipeline for data preprocessing: data filtering, data labeling and data splitting, which are detailed below.

#### Data Filtering

We filter the raw patient claims with the following criteria: (1) Keep Main Hospital (MH) claims with status ‘Approved’; (2) Keep patients who have ever been diagnosed with at least one of 491.*, 492.*, and 496.* in MH DX claims; (3) Keep patients who are at least 40 years old; (4) Keep patients with decided gender; (5) Keep patients with at least one Inpatient MH claim in the entire history; (6) Keep patients with observation history of at least 60 days; (7) Keep patients with at least one pharmacy claim in the entire history. The detailed patient information before and after each filtering criterion can be found in the Supplementary Material.

#### Data Labeling

In order to build the predictive model, we further label each patient hospital admission as either index admission or readmission. Specifically, a hospital readmission is when a patient who had been discharged from a hospital is admitted again to the same or a different hospital within 30 days. The original hospital admission is referred to as index admission, and the subsequent admission is referred to as readmission. We further have the following inclusion criteria for index admissions in our study.

1. The patient has enrollment information for at least 30 days after the discharge. This is necessary to gaurantee that readmissions within 30 days can be tracked.
2. The patient was enrolled for 12 months prior to the index admission. This is necessary to gather adequate clinical information for accurate risk adjustment.

One issue we need to deal with is hospital transfer. A hospital transfer is the case in which a patient is discharged from a hospital and admitted to another hospital at the same day. Therefore, we have 6 classes of hospital admissions in total: index admission, index transfer (the patient is transferred at the same day of the index admission), index final (this is the last stop of the transfer), readmission, readmission transfer, and readmission final. The numbers of all 6 kinds of admissions are provided in the supplemental material. Finally, we get 67,771 index admissions (i.e, index and index_final), among which 10,265 (15.15%) samples are followed by a 30-day readmission. There are 27,138 patients involved in these hospital stays. We summarize the statistical characteristics of the overall samples in Table 2.

**Table 2:** Summary statistics of the 67,771 patients.

Variables	Mean	Std	Min	Max	Variables	Mean	Std	Min	Max
Age	72.10	11.83	29	99	Readm_trans	0.01	0.11	0	6
Gender	0.50	0.50	0	1	Readm_final	0.01	0.09	0	2
LOS	5.00	6.21	0	389	LACE_L	3.43	1.49	0	7
LOAS	9.74	12.28	0	404	LACE_A	2.04	1.40	0	3
NOA	1.89	1.36	1	16	LACE_C	2.73	1.15	0	4
NOAA	55.17	38.07	1	493	LACE_E	1.85	1.40	0	4
Index	1.53	0.90	0	7	HOS_Proc	0	0	0	0
Index_trans	0.10	0.36	0	7	HOS_LOS	0.78	0.97	0	2
Index_final	0.09	0.30	0	3	HOS_NOAD	1.00	1.19	0	5
Readm	0.16	0.56	0	13	HOS_Index	0.68	0.47	0	1

#### Data Splitting

We apply five-fold cross validation on all 27,138 patients to evaluate the performance of the investigated approaches. Note that we cannot apply five-fold cross validation on discharges, because if one patient has multiple discharges, it is possible that some of these discharges are in training set while some are in validation set. This may produce overly optimistic performance due to label leaking.

### Traditional Methods

We implemented seven different traditional machine learning models with different of feature sets as introduced in the Methods Section. The results are summarized below.

#### Knowledge-driven features

**Figure 2:**
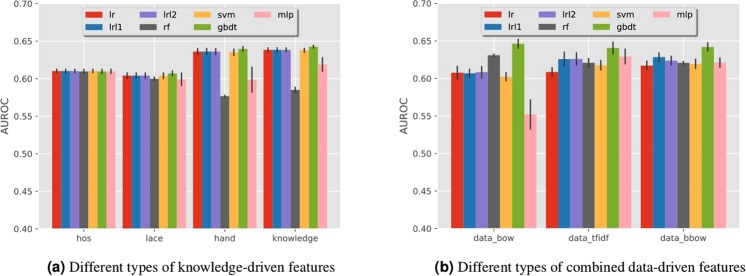
AUC performance achieved by predictive models with different types of features and machine learning models. In (**a**), ‘hos’, ‘lace’, ‘hand’, ‘knowledge’ represent HOSPITAL score, LACE index, handcrafted feature, and the combination of all these three kinds of features. For the legend, ‘lr’, ‘lrl1’, ‘lrl1’, ‘rf’, ‘svm’, ‘gbdt’, ‘mlp’ represent logistic regression, logistic regression with L1 penalty, logistic regression with L2 penalty, random forest, support vector machine, gradient boosting decision tree, and multi-layer perceptron. The same naming convention is also applied in the legends of the follow-up figures. In (**b**), COPD readmission prediction performance with combined data -driven features. For the x-axis, ‘data_bow’, ‘data_tfidf’ and ‘data_bbow’ represent BoW, TFIDF and BBoW features.

The prediction performance in terms of Area Under the r eceiver o perating c haracteristic (ROC) Curve (AUC) with knowledge-driven features are shown in Fig. 2(a). These features are extracted from the one-year history prior to the discharge of the index admission. We can observe that:

1. The two baseline methods, HOSPITAL score and LACE index, have similar performance with AUC around 0.60, and HOSPITAL score is slightly better.
2. Our handcrafted features can produce better performance than the two baseline methods.
3. The combined knowledge-driven features lead to the best performance, with the mean AUC of 0.643 using the GBDT classifier.

To better understand knowledge-driven features, we further investigate the trained logistic regression model. We record the coefficients of all predictors in Table 3. We can find that older age, male gender, longer length of stay, and more admissions in previous year will increase the risk of readmission. It is interesting to notice that a larger number of index_trans in the previous year will decrease the risk. The reason could be that more hospital transfers lead to better patient care. The LACE index features and HOSPITAL score features have positive relationship with readmission risk, except HOS_Proc, HOS_index.

**Table 3:** Coefficients of knowledge-driven features in LR model and LR model with ${\ell }_{1}$ penalty.

	Age	Gender	LOS	LOAS	NOA	NOAA	Index
LR	0.0032	0.0996	0.0174	−0.0089	0.0861	0.0056	0.0002
LR_l1	0.0026	0.0987	0.0172	−0.0087	0.0847	0.0056	0.0
	**Index_trans**	**Index_final**	**Readm**	**Readm_trans**	**Readm_final**	**LACE_L**	**LACE_A**
LR	−0.1364	0.1081	0.0893	0.2166	−0.1918	0.0737	0.0213
LR_l1	−0.1238	0.0914	0.0885	0.1177	−0.0677	0.0732	0.0235
	**LACE_C**	**LACE_E**	**HOS_Proc**	**HOS_LOS**	**HOS_NOAD**	**HOS_Index**	**Intercept**
LR	0.0444	0.0761	0.0	0.0536	0.0747	0.0071	−1.5246
LR_l1	0.0493	0.0761	0.0	0.0537	0.0753	0.0	−1.4950

#### Data-driven features

For data-driven features, we combine the grouped diagnosis, grouped procedure, grouped pharmacy and location codes together to obtain the combined data-driven features, whose performances are summarized in Fig. 2(b). From the figure we can observe that the best mean AUC value is around 0.646, which can be obtained from the BoW representation using GBDT classifier.

**Figure 3:**
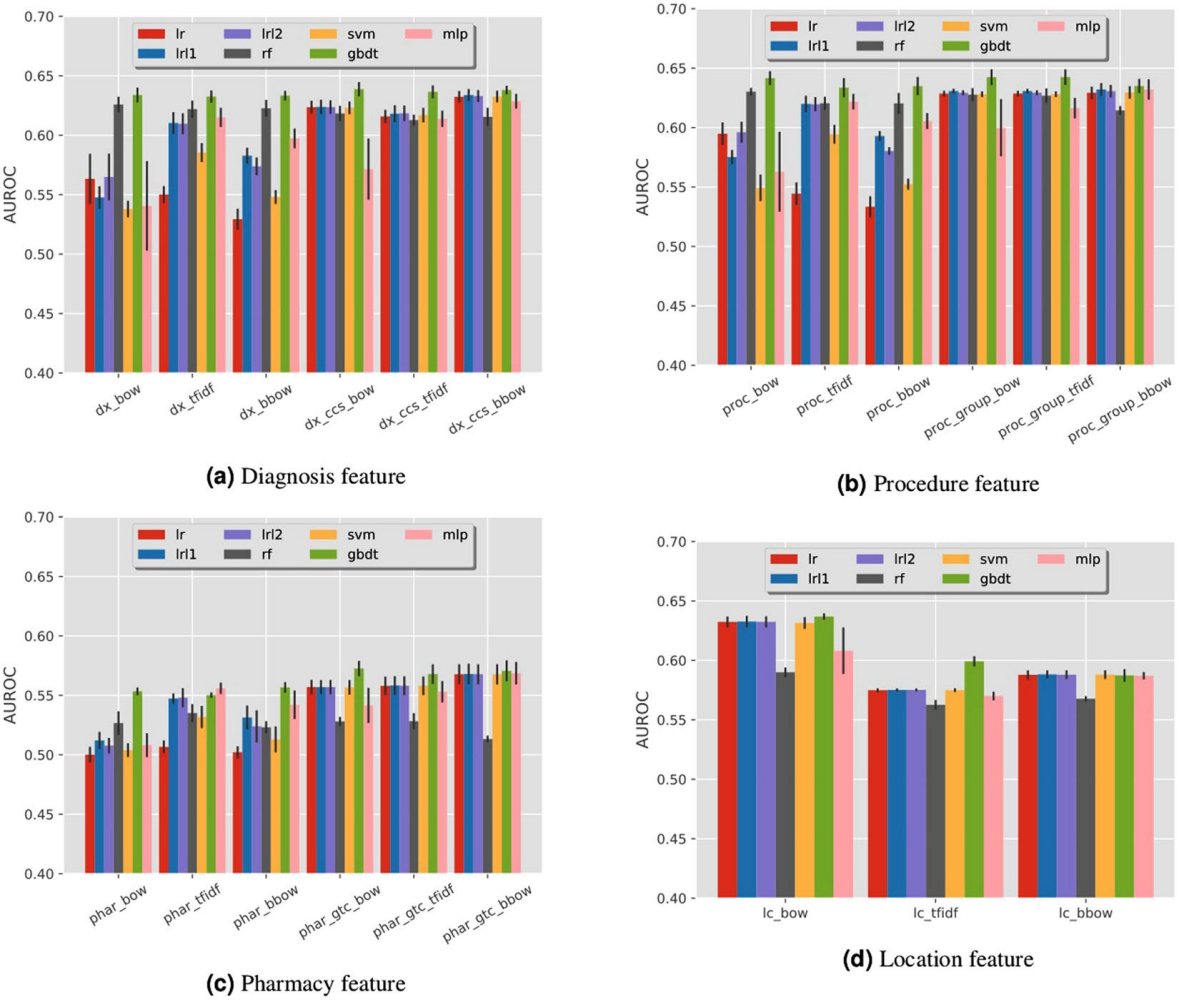
Comparisons of the predictive performance of different types of data-driven features on COPD readmission. In (**a**), ‘dx_bow’, ‘dx_tfidf’, ‘dx_bbow’ represent BoW, TFIDF and BBoW feature for diagnosis records. ‘dx_ccs_bow’, ‘dx_ccs_tfidf’, ‘dx_ccs_bbow’ represent Bow, TFIDF and BBoW feature for grouped diagnosis codes using CCS hierarchy. The same naming convention also applies to (**b**–**d**).

We further explore how different types of data -driven features influence the prediction performance. These features are extracted from the one-year history prior to the discharge date of the index admission. The results are shown in Fig. 3, from which we can observe that:

1. The grouped codes (e.g., diagnosis codes grouped by CCS) can produce better performances than the original raw codes. This is potentially due to the high dimensionality of the raw codes, which results in highly sparse feature representations. Grouping the codes can greatly reduce the dimensionality and thus increase the density of the feature vector.
2. Comparing with other features, diagnosis and procedure are more useful to the readmission prediction task, while the pharmacy feature is not very informative.
3. The GBDT classifier generally achieves the best performance among the seven traditional classifiers for most of the features.

#### The Effect of Observation Window Lengths

We also explored how the observation window length will affect the readmission prediction performance. We compared the performance of one-year observation window against the full-history. All knowledge- and data-driven features are concatenated. The results are summarized in Table 4, from which we can observe that:

1. For the one-year observation window, we can obtain the best AUC of 0.653 using GBDT, which is better than knowledge-or data-driven features alone.
2. Increasing the observation window from one year to full history barely improves the performance of GBDT, while most of other models get obvious improvements.

**Table 4:** Prediction performance for comprehensive features extracted from one-year history and from full history.

	LR	LR_l1	LR_l2	RF	SVM	GBDT	MLP
One year	0.617	0.616	0.617	0.636	0.612	0.653	0.571
Full history	0.635	0.644	0.645	0.624	0.643	0.654	0.627

### Deep Learning Methods

For deep learning experiments, we focus on the impact of different time fusion and embedding strategies.

#### Time Fusion Strategies

**Figure 4:**
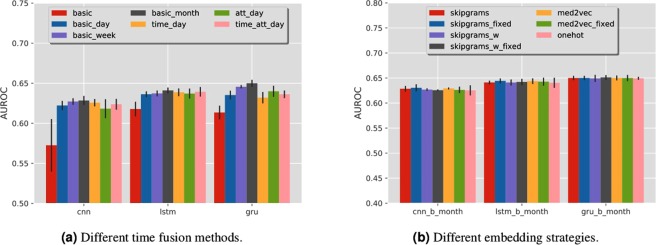
Performance comparison among different time fusion and different embedding strategies. In (**a**), ‘basic’ indicates the most basic model where we use the sequence input without any time weighting or attention mechanisms. ‘basic_day’, ‘basic_week’ and ‘basic_month’ indicate the model using matrix input with regular time interval, whose time granularity is day, week and month. ‘time_day’, ‘att_day’, ‘time_att_day’ indicate the model using matrix input of irregular time interval, plus the time weighting layer, attention weighting layer, and both layers. For all models, we adopt Word2Vec embedding, and let the embedding layer be trainable when training the deep models. In (**b**),’skipgrams’,’skipgrams_w’ and’med2vec’ indicate the models using embedding matrix learned by Skip-grams model, the time weighted Skip-grams model, and the Med2vec model. The suffix’_fixed’ means that we keep the parameters in the embedding layer fixed during training.’one-hot’ indicates the model simply uses the one-hot embedding layer.

We compare the performance of different time fusion methods in Fig. 4a, from which we can observe that:

1. The basic sequence classification without considering time information generates the worst performance. This means that considering the exact event timestamps can indeed improve the prediction performance.
2. Matrix representation with regular time intervals performs better than sequence representation.
3. Matrix representation with irregular time interval combined with event attentions does not necessarily improve the prediction performance.
4. If we use a coarse time granularity, for example by week or month instead of by day in matrix representations, the prediction AUC can be improved. The best performance of AUC 0.650 is achieved by GRU model based on matrix representation by month.

#### Embedding Strategies

We also explored the impact of different embedding strategies. We used the matrix representation with regular time intervals aggregated by month. The performance of using different embedding strategies is summarized in Fig. 4(b), from which we do not observe significant differences across the performances of different embedding strategies.

## Discussions

From our investigations above on the task of readmission risk prediction for COPD patients based on patient claims data, we have the following observations.

1. *Knowledge is powerful*. Similar to what has been observed in Rajkomar *et al*.^19^, simple models based on clinical knowledge, such as LACE and Hospital Score, work pretty well in reality. We also expanded the knowledge-driven features used in these two models to a broader set (see the handcrafted features in Table 1), which can further improve the prediction performance in terms of AUC (from 0.61 to 0.64). Comparing with data-driven features, those knowledge-driven features are highly interpretable and generalizable.
2. *Data-driven features are helpful*. With the data-driven features, we can improve the prediction performance (from 0.64 to 0.65). Combining the knowledge- and data-driven features leads to the best prediction performance (around 0.653).
3. *GDBT is powerful*. Comparing with other traditional machine learning models, GBDT can achieve better performance almost across all different experimental settings, and it obtained the best performance with the combination of both knowledge- and data-driven features.
4. *Longer history barely helps*. We do not observe much differences on the prediction performance on patient records with one-year observation window or full-history. This observation also explains implicitly why only one year history was used in both LACE and HOSPITAL Score models.
5. *Deep learning barely helps*. We have systematically investigated the performance of various deep learning models, including the variants of CNN and RNN with different representation, embedding and time-sensitive strategies. However, the best performance achieved among them is on par with the best performance of GDBT (around 0.65). The same phenomenon is also observed in Rajkomar *et al*.^19^.

With these observations, we can conclude that predicting the risk of hospital readmission is difficult based on only claims data. Machine learning models can benefit when combining patient data with clinical knowledge. This is potentially be explained from the following aspects.

1. Medicine has been a research discipline with long history. The medical knowledge people accumulated from clinical practice are invaluable and powerful.
2. Unlike other application domains such as computer vision and natural language processing, where deep learning models have been shown to be very powerful, medical problems are much more complicated and with less available training samples. This means that it is difficult to have a ‘sufficiently large’ patient dataset to train a very good machine learning model. In this case, incorporating domain knowledge into the model building process is of vital importance, and complex models do not necessarily lead to better performance as they need even more training samples.
3. The information contained in patient claims records may not be sufficient for building good hospital readmission risk prediction models. Some important and relevant clinical features, such as GOLD severity grade, are not available. More comprehensive and finer granular patient data, such as electronic health records, could be potentially more helpful.
4. Our claims data lacks mortality information of patients. In fact, hospital readmission risk and death risk are competing clinical risks, since patients that die after discharge cannot be readmitted, which makes the risk of readmission and death after discharge are often negatively linked. However, there could be some common causes for both risks (e.g., condition exacerbation), which could confuse the predictive models.

## Conclusion

We conducted a comprehensive study on predictive modeling of the 30 day readmission risk of COPD patients based on their claims records with various machine learning models. We constructed both knowledge- and data-driven features from the patients’ claim records to train the predictive models. Both traditional and modern machine learning models are investigated. The results showed that the combination of both knowledge and data driven features can lead to the best prediction performance, and complicated models such as deep learning can barely improvement the performance. Our studies verify the importance of medical knowledge in the predictive modeling process, as well as the demands for better patient data.

## Supplementary information

Supplementary Material
