# Supplementary material for: Predictive Modeling of the Hospital Readmission Risk from Patients’ Claims Data Using Machine Learning: A Case Study on COPD
Source: Sci Rep. 2019 Feb 20;9:2362. doi: 10.1038/s41598-019-39071-y (PMC6382784; doi:10.1038/s41598-019-39071-y)
Supplement: Supplementary file 1 — Supplementary Material [file 41598_2019_39071_MOESM1_ESM.pdf]

Predictive Modeling of the Hospital Readmission Risk from Patients' Claims Data Using Machine Learning: A Case Study on COPD

Xu Min[1,2], Bin Yu[3], and Fei Wang[1,*]

[1]Department of Healthcare Policy and Research, Weill Cornell Medicine. New York. NY. USA.
[2]Department of Computer Science and Technology, Institute for Artificial Intelligence, Tsinghua-Fuzhou Institute for Data Technology, and Bioinformatics Division, BNRist, Tsinghua University. Beijing. China.
[3]American Air Liquide, Newark. DE. USA
[*]Corresponding Author. Email: few2001@med.cornell.edu

Supplemental Information

Formally, the Skip-grams model is to minimize the loss function

$$\mathscr{L}(\theta) = \frac{1}{T}\sum_{t=1}^{T}\sum_{-m\leq j\leq m} -\log p(w_{t+j}|w_t), \tag{1}$$

where the probability of predicting an outside word using a center word is defined as

$$p(o|c) = \frac{\exp(u_o^T v_c)}{\sum_{i=1}^{V}\exp(u_i^T v_c)}. \tag{2}$$

We use the vector representations $v_c \in \mathbb{R}^d$ and $u_o \in \mathbb{R}^d$, for the center word and the outside word, respectively in our model. Hence we have $\theta = [v_1, v_2, \cdots, v_V, u_1, u_2, \cdots, u_V] \in \mathbb{R}^{d\times 2V}$, which is all the parameters to be learned in the model.

However, the normalization factor of the softmax function is too computationally expensive. Instead, we train a binary logistic regression for a true pair (center word and outside word in its context window) and a couple of random pairs (center word and a random word in the vocabulary), which is called negative sampling. Thus, the loss function is

$$\mathscr{L}(\theta) = \frac{1}{T}\sum_{t=1}^{T}\mathscr{L}_t(\theta), \tag{3}$$

$$\mathscr{L}_t(\theta) = -\sum_{o\in\text{context}}\left\{\log\sigma(u_o^T v_c) + \sum_{j=1}^{k}\mathbb{E}_{j\sim P(w)}[\log\sigma(-u_j^T v_c)]\right\}. \tag{4}$$

As is visualized in Figure 2, the Skip-gram model is actually a shallow one-layer neural network model.

We adopted modifications on Skip-gram model.

1. The first is to use a time window instead of a context window to generate event contexts. This modification is straightforward.

$$\mathscr{L}_t(\theta) = -\sum_{\Delta_{o,c}\leq\Delta}\left\{\log\sigma(u_o^T v_c) + \sum_{j\sim P(w)}\log\sigma(-u_j^T v_c)\right\}, \tag{5}$$

where $\Delta_{o,c}$ is the in-between time gap of the two medical codes, and Δ is the length of time window.

2. The second is to weight the event pairs according to the time gap of the two them, such that those pairs whose codes occurring closer in time can be assigned with higher weights. To incorporate time information, those pairs whose codes occurring closer in time can be assigned with higher weights in the loss function $\mathscr{L}(\theta)$.

$$\mathscr{L}_t(\theta) = -\sum_{\Delta_{o,c}\leq\Delta}\left\{w(\Delta_{o,c})\log\sigma(u_o^T v_c) + \sum_{j\sim P(w)}\log\sigma(-u_j^T v_c)\right\}, \tag{6}$$

where $w(\Delta_{o,c})$ is a time window function. For example, we can define $w(\Delta_{o,c}) = e^{-a\Delta_{o,c}}$, if we apply an Exponential or Poisson window, or $w(\Delta_{o,c}) = a(\Delta - \Delta_{o,c})$ if we apply a Triangular window. We can also use a Hamming window, a Gaussian window, and so on.

Following the medical concept embedding and the time fusion methods just introduced, we are now ready to introduce different architectures of the deep learning models we use to predict COPD readmissions. Suppose we have a code sequence $c_1, c_2, \cdots, c_L$ or a code matrix $\mathbf{C} \in \mathbb{R}^{L\times V}$ as input, we first embed it into a weight matrix $\mathbf{W} = \mathbf{CU}$, where $\mathbf{U} \in \mathbb{R}^{V\times d}$ is an embedding matrix which embeds V unique codes into a d-dimensional vector space. Hence, we obtain an embedded matrix $\mathbf{W} \in \mathbb{R}^{L\times d}$, each row of which, namely w_i, is a d-dimensional embedded vector. If the time interval of the code matrix $\mathbf{C}$ is irregular, we need to add a time weighting layer. We calculate the time weight sequence $d_i \propto softmax(\lambda \cdot t_i)$ according to the time sequence input. Apart from using the time weighting layer to incorporate timestamp information, we can also apply attention mechanism on the the embedding matrix to pay more weight on those medical concepts of more importance. The attention weight can be computed using a softmax function $a_i \propto softmax(\beta^T \mathbf{w}_i)$, where β is a reference vector to be learned during training model. This attention weight a_i tells us how much attention we should put on the code c_i. We can choose to use either two kinds of weights, or combine both of them, or use neither, that is to say, we can get a new weighted vector $\mathbf{w}'_i = d_i\mathbf{w}_i$, or $\mathbf{w}'_i = a_i\mathbf{w}_i$, or $\mathbf{w}'_i = d_i a_i \mathbf{w}_i$, or simply $\mathbf{w}'_i = \mathbf{w}_i$. Thus, based on the new matrix $\mathbf{W}'$, we can now use either CNN, LSTM or GRU to extract features, and then use fully-connected layers with softmax layer to predict the final probability of readmission. We visualize our model in Figure 3 in Supplemental Figures.

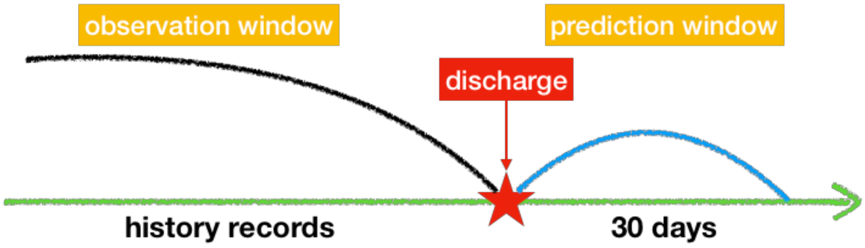

Figure 1. Visualization of observation window and prediction window.

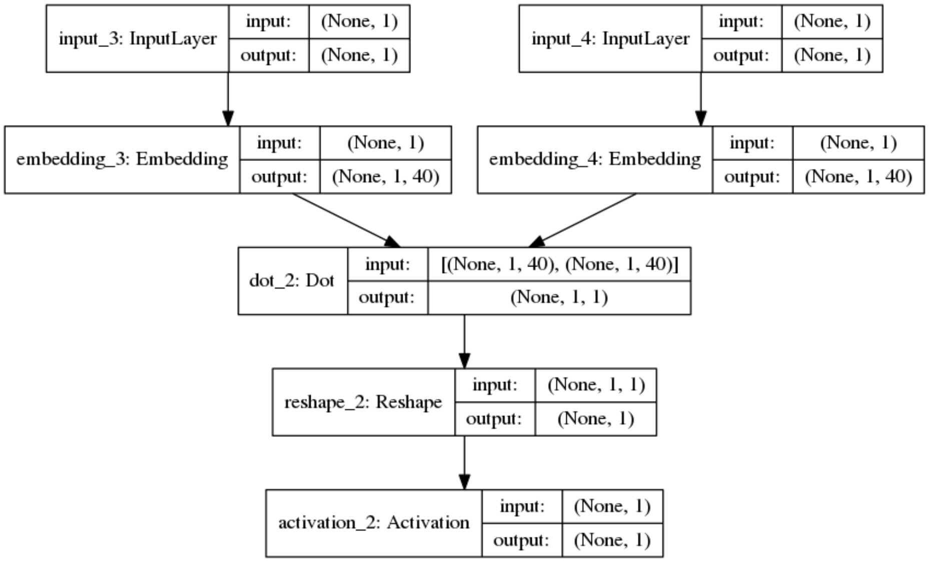

Figure 2. Skip-grams architecture.

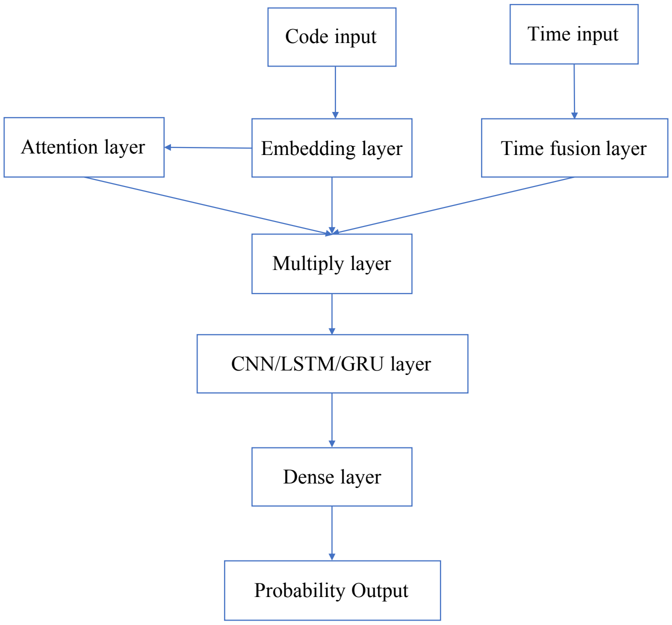

Figure 3. The general architecture of the proposed deep learning models.

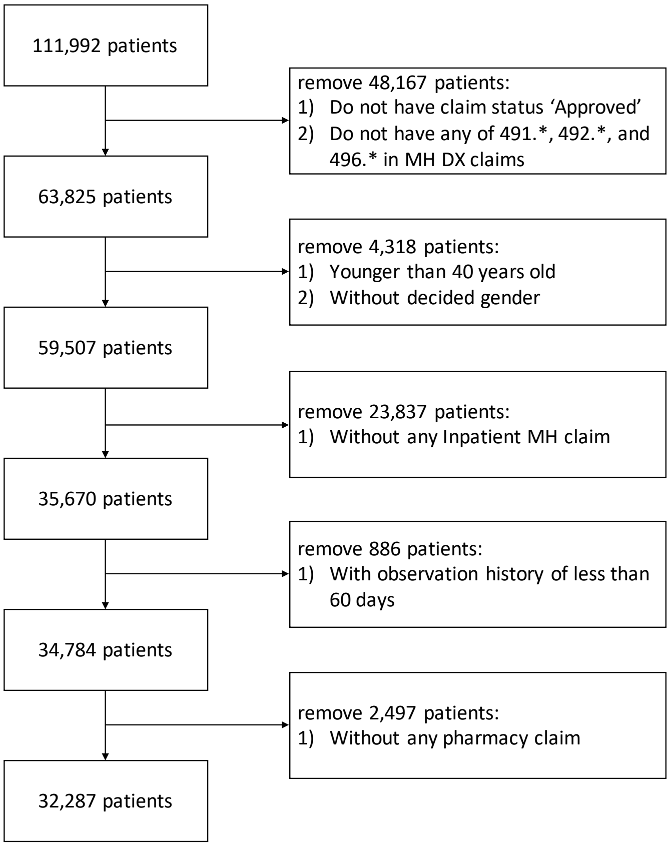

Figure 4. The flowchart of patient sample creation.

data table	attributes
Demographics	Pt_ID, Age, Gender, First_Act_Day, Last_Act_Day, First_Qualify_Day, Index_Day_Year.
Pharmacy Claims	Pt_ID, PClaim_ID, Written_Day, Filled_Day, Generic_Therapeutic_Class_Code, Generic_Therapeutic_Class_Desc, NDC_CODE, etc.
Medical/Hospital Claims	Pt_ID, MHClaim_ID, Admission_Day, Discharge_Day, Program, Claim_Type, Medical_Claim_Class, Loc_Code, Loc_Desc, Claim_Status.
Medical/Hospital Claims Diagnosis	Pt_ID, MHClaim_ID, ICD_9_Code, ICD_9_Code_Precedence, ICD_9_Day
Medical/Hospital Claims Procedures	Pt_ID, MHClaim_ID, Procedure_Code, Procedure_Code_Modifier, Referral_ID, Procedure_Day, Procedure_Quantity.

Table 1. Geisinger Claims Data

# of records	before filtering	after filtering
patient	111,992	32,287
MH claim	14,578,751	6,713,724
MH DX	50,441,847	25,854,159
MH PROC	39,379,681	19,776,740
PHAR claim	16,680,306	8,471,026

Table 2. Overview of filtering data

admission type	number
index	82,156
index_trans	5,451
index_final	4,863
readm	17,422
readm_trans	1,051
readm_final	932
total	111875

Table 3. Number of admissions

abbreviation	meaning
LR	Logistic regression
LR_l1	Logistic regression with ℓ_1 penalty
LR_l2	Logistic regression with ℓ_2 penalty
RF	Random Forest
SVM	Support Vector Machine
GBDT	Gradient Boosting Decision Tree
MLP	Multi-Layer Perceptron

Table 4. Taxonomy of the methods used in this paper
